# VUV absorption spectra of water and nitrous oxide by a double-duty differentially pumped gas filter

**DOI:** 10.1107/S1600577524005423

**Published:** 2024-07-23

**Authors:** Andras Bodi, Jonas Knurr, Patrick Ascher, Patrick Hemberger, Christoph Bostedt, Andre Al Haddad

**Affiliations:** a Paul Scherrer Institute 5232Villigen-PSI Switzerland; University of Essex, United Kingdom

**Keywords:** vacuum ultraviolet, absorption spectroscopy, differential pumping, gas phase

## Abstract

The differentially pumped rare-gas filter at the end of the VUV beamline of the Swiss Light Source has been adapted to house a windowless absorption cell for gases. Absorption spectra can be recorded from 7 eV to up to 21 eV photon energies routinely, as shown by a new water and nitrous oxide absorption spectrum. By and large, the spectra agree with previously published ones both in terms of resonance energies and absorption cross sections, but that of N_2_O exhibits a small shift in the  band and tentative fine structures that have not yet been fully described. This setup will facilitate the measurement of absorption spectra in the VUV above the absorption edge of LiF and MgF_2_ windows. It will also allow us to carry out condensed-phase measurements on thin liquid sheets and solid films. Further development options are discussed, including the recording of temperature-dependent absorption spectra, a stationary gas cell for calibration measurements, and the improvement of the photon energy resolution.

## Abstract 2

The differentially pumped gas filter of the VUV beamline at the SLS is adapted to record VUV absorption spectra of gaseous samples over a broad photon energy range. Water and nitrous oxide spectra are recorded in proof-of-principle measurements.

## 1. Introduction

Photoabsorption, in which condensed matter or isolated species absorb photons and transition to higher-energy states, underpins a multitude of natural and synthetic systems, most notably vision, photosynthesis and photovoltaics. While these rely on the spectral range with the maximum intensity of solar radiation, *i.e.* on visible light, absorption spectroscopy spans the entire electromagnetic spectrum from microwaves to X-rays. Microwave spectroscopy reveals molecular rotation and, thus, geometries. Room-temperature black-body radiation peaks in the infrared, which is why infrared absorption drives the greenhouse effect. Infrared spectroscopy is a structural analytical tool in the energy range of vibrational excitation. Visible (vis), ultraviolet (UV) and vacuum ultraviolet (VUV) photons have energies that allow for electronic excitation of valence states, potentially leading to ionization above *h*ν > 5 eV photon energy (λ < 250 nm). The UV-vis as well as VUV absorption spectra of gaseous molecules of atmospheric interest have been compiled in the eminently useful MPI-Mainz UV/VIS Spectral Atlas (Keller-Rudek *et al.*, 2013 ▸). X-ray absorption involves core-excitation states. Thanks to synchrotron and free-electron laser facilities, tender and hard X-ray experiments have provided a wealth of structural information on condensed-state samples (Willmott, 2019 ▸).

VUV absorption spectra of gaseous samples give insights into electronic excitations to high-lying valence states, allow the identification of Rydberg states and their progressions, and VUV absorption may lead to photoionization above the ionization energy. In small molecules, vibrational progressions can often be resolved and assigned. Above the ionization energy, the ratio of the photoionization and the photoabsorption cross sections yields the photoionization quantum efficiency, which reveals fundamental mechanistic details. For instance, the water threshold photoelectron spectrum (Truong *et al.*, 2009 ▸) and the breakdown diagram of the water cation (Bodi *et al.*, 2014 ▸) show that the threshold photoionization cross section jumps fourfold at the H-loss dissociative ionization onset. With no comparable jump discernible in the photoabsorption oscillator strengths (Gürtler *et al.*, 1977 ▸), this implies that the branching ratio to threshold photoionization changes suddenly as the dissociative ionization channel opens up. This identifies a photoionization process beyond the sudden approximation (Melania Oana & Krylov, 2007 ▸; Bodi *et al.*, 2017 ▸), which is an important aspect regarding photoionization cross-section calculations. VUV absorption spectra are also connected to resonance-enhanced multiphoton ionization experiments, in which the neutral states seen in the absorption spectrum act as intermediate states, resonantly excited in multiphoton ionization (Ashfold *et al.*, 1984 ▸; Yang *et al.*, 2010 ▸). Beyond fundamental insights into electronic and vibronic structure as well as photoionization transitions, VUV absorption also plays a direct role in environments with a strong VUV radiation field. For instance, black-body radiation peaks in the VUV range at temperatures around 20000 K. Therefore, VUV absorption plays an important role in plasma physics and in the radiation field of blue stars (Cruz-Diaz *et al.*, 2014 ▸). Furthermore, VUV-induced chemistry may also play a role at the edge of the atmosphere (Gao, 2021 ▸). In fact, relatively recent data on water vapor in the 6.23–6.85 eV energy range, recorded using a hydrogen discharge lamp, continue to influence terrestrial and extraterrestrial atmospheric models (Parkinson & Yoshino, 2003 ▸). Generally, the weak absorption of solar radiation by water vapor in the near-UV range between 3.1 and 6.2 eV at characteristic cross sections of 10^−24^ cm^2^ is a poorly understood and neglected aspect in atmospheric radiation models leading to errors in the range 0.2–0.8 W m^−2^ (Makogon *et al.*, 2013 ▸; Pei *et al.*, 2019 ▸).

In hydro­carbons and oxygenates, VUV absorption is associated with the excitation of σ and *n* electrons into σ* and Rydberg orbitals. As σ orbitals are sensitive to the molecular structure, VUV absorption spectra can contribute to the identification of polyatomic molecules relevant in low-temperature combustion and atmospheric chemistry (Doner *et al.*, 2022 ▸). Electronic structure theory can be applied to compute these transitions, *e.g.* for oxygenates (Bralick *et al.*, 2023 ▸), whereas Franck–Condon modeling and on-the-fly *ab initio* semiclassical approaches can be used to simulate the vibrational structure of the spectrum (Patoz *et al.*, 2018 ▸).

Measuring VUV absorption spectra in the gas phase can be challenging. There are few laboratory-based light sources that deliver sufficient flux in the VUV. Historically, Hopfield-continuum light sources were used (Cook & Metzger, 1964 ▸), for instance to record the absorption spectrum of nitrous oxide (N_2_O) in the 12.4–20.7 eV range (Cook *et al.*, 1968 ▸). Deuterium lamps can be used in the 5–10 eV range to enable quantitative isomer-resolved speciation measurements of functionalized hydro­carbons in combustion chemistry (Christianson *et al.*, 2021 ▸). Machine learning models were also proposed for binary molecular classification using VUV absorption spectra obtained in the laboratory (Doner *et al.*, 2023 ▸). Nevertheless, broadband VUV absorption studies have primarily been carried out at synchrotron light sources. These deliver plenty of VUV flux but have stringent vacuum requirements to protect the accelerator. A differential pumping scheme was used together with a 100 nm zapon (cellulose nitrate) film to record the absorption cross sections of rare gases at the Synchrotron Radiation Source (SRS) at Daresbury Laboratory (West & Marr, 1976 ▸). Later, a capillary beam guide was utilized for differential pumping when recording the nitrous oxide absorption spectrum in the 12.9–25.6 eV range at the SRS (Shaw *et al.*, 1992 ▸). Further windowless differentially pumped absorption cells were used to record VUV photoabsorption spectra, for example for water vapor in the 12.4–20.7 eV range at DESY (Gürtler *et al.*, 1977 ▸) and for nitrous oxide in the 5.9–12.4 eV range at the National Synchrotron Radiation Research Center (NSRRC) at Hsinchu (Nee *et al.*, 1999 ▸). Fillion *et al.* reported a high-resolution water vapor absorption spectrum between 10.9 and 12.0 eV at the LURE-Super-ACO synchrotron (Fillion *et al.*, 2004 ▸). Nonetheless, these sources have either been decommissioned or are nearing their end of life, which means that experimental stations where broadband VUV absorption spectra can be recorded above 11 eV (λ < 113 nm) are virtually nonexistent. A notable exception is the state-of-the-art Fourier transform spectrometer at the DESIRS beamline of SOLEIL, which allows for the measurement of absorption cross sections in a molecular beam and at room temperature in the 4–30 eV photon energy range with a limiting resolution of 0.01 meV (10^−4^ nm at 10 eV) (de Oliveira *et al.*, 2016 ▸). This setup has been employed to record the high-resolution absorption spectrum of room-temperature and jet-cooled ammonia between 7.32 and 11.53 eV (Pratt *et al.*, 2023 ▸) and that of two di­hydro­furan isomers in the 5.5–9.4 eV photon energy range (Röder *et al.*, 2020 ▸).

Windowed absorption cells can only reach energies up to 11.3 eV (λ > 110 nm) in the VUV because of the absorption edge of the window material, typically LiF or MgF_2_. They are more broadly available and have been used to record a multitude of VUV absorption spectra. Also at the Daresbury light source, a windowed setup was used to measure the VUV absorption spectrum of ozone (Mason *et al.*, 1996 ▸). Duflot *et al.* reviewed synchrotron-based VUV photoabsorption studies of gaseous samples (Duflot *et al.*, 2019 ▸), focusing on work done at the ASTRID synchrotron at Aarhus, Denmark, using a windowed absorption cell (Eden *et al.*, 2006 ▸). Mota *et al.* recorded the absorption cross section of water at a resolution of ∼4 meV in the 6–11 eV photon energy range there (Mota *et al.*, 2005 ▸). More recently, the VUV absorption spectrum of ammonia was recorded in the 5.4–10.8 eV range by Limão-Vieira *et al.* (2019 ▸), and Ovad *et al.* recorded the VUV absorption spectrum of C_4_F_7_N, a dielectric gas and SF_6_ replacement candidate, at the updated ASTRID-2 storage ring also in a windowed cell (Ovad *et al.*, 2023 ▸). Finally, VUV absorption spectra have been recorded at the AMO beamline of the INDUS(-2) synchrotron with 4 meV resolution and the Rydberg progressions assigned, for example, for *N*,*N*-di­methyl­formamide in the 5.6–9.9 eV energy range (Shastri *et al.*, 2017 ▸) and for nitro­methane in the 5.4–11.8 eV range (Shastri *et al.*, 2021 ▸). The latter study has since been followed up by further absorption work from ASTRID-2 (Dalagnol *et al.*, 2023 ▸).

Alternatively to photoabsorption, electron energy-loss dipole (e,e) spectroscopy can be used to measure oscillator strengths that relate to the absorption cross section directly. The main benefit of (e,e) spectroscopy is its easy implementation. However, it suffers from constraints in spectral resolution, and the accuracy of the data obtained is contingent on the validity of the dipole approximation, which may not always hold. Electron impact coincidence simulation techniques were applied to study absorption, ionization and fragmentation of water in the 10–60 eV energy range using 3.5 keV electrons (Tan *et al.*, 1978 ▸). Later, (e,e) spectroscopy yielded water cross sections in the 6–200 eV energy range using 8 keV electrons (Chan *et al.*, 1993 ▸). Notwithstanding the broad energy range, the 1 eV resolution means that nearby bands overlap, and the vibrational fine structure of the spectrum remains out of reach. Thus, (e,e) spectroscopy is a worthwhile complementary tool, but it rarely reveals the full richness of the VUV absorption spectrum. For that, direct photoabsorption measurements remain indispensable.

The VUV beamline at the Swiss Light Source (SLS) has a bending magnet source, which requires a large horizontal acceptance and results in a relatively large divergence of the beam at 8 mrad (h) × 4 mrad (v). To provide high-harmonic-free VUV radiation, VUV beamlines often rely on thin films, as recently implemented at FinEstBeAMS at MAX IV (Chernenko *et al.*, 2021 ▸). However, particularly in the 10–21 eV range, rare-gas filters are used, which are almost fully transparent below the ionization energy of the rare gas and let virtually no light pass above it. The divergent bending magnet radiation means that the light beam cannot be led through a capillary, as is often done at undulator light sources, such as at the Chemical Dynamics beamline at the ALS and DESIRS at SOLEIL (Suits *et al.*, 1995 ▸; Mercier *et al.*, 2000 ▸). Furthermore, in the absence of re-focusing optics to minimize the number of optical elements and maximize the usable flux, the gas filter had to be placed near the experimental station at the end of the beamline. Satisfying these requirements, a differentially pumped filter was developed with eight chambers. Its absorption chamber, with an optical length of 10 cm, can accept ∼30 mbar of rare gas without compromising the 10^−7^ mbar pressure in the transfer line. At a rare-gas pressure of 10 mbar, the suppression factor for the high-harmonic radiation of the grating was measured to be ∼10^6^ (Johnson *et al.*, 2009 ▸). The gas filter is, thus, effectively a differentially pumped, windowless absorption cell, which has motivated us to evaluate it in VUV absorption measurements above the MgF_2_ edge. We chose two atmospherically relevant and well characterized gaseous samples – water and nitrous oxide – and will compare the spectra with the literature results. In addition, we have constructed the absorption cell to allow for condensed phases, such as a thin film or free-flowing liquid sheet targets (Koralek *et al.*, 2018 ▸).

## 2. Experimental

**Figure 1:**
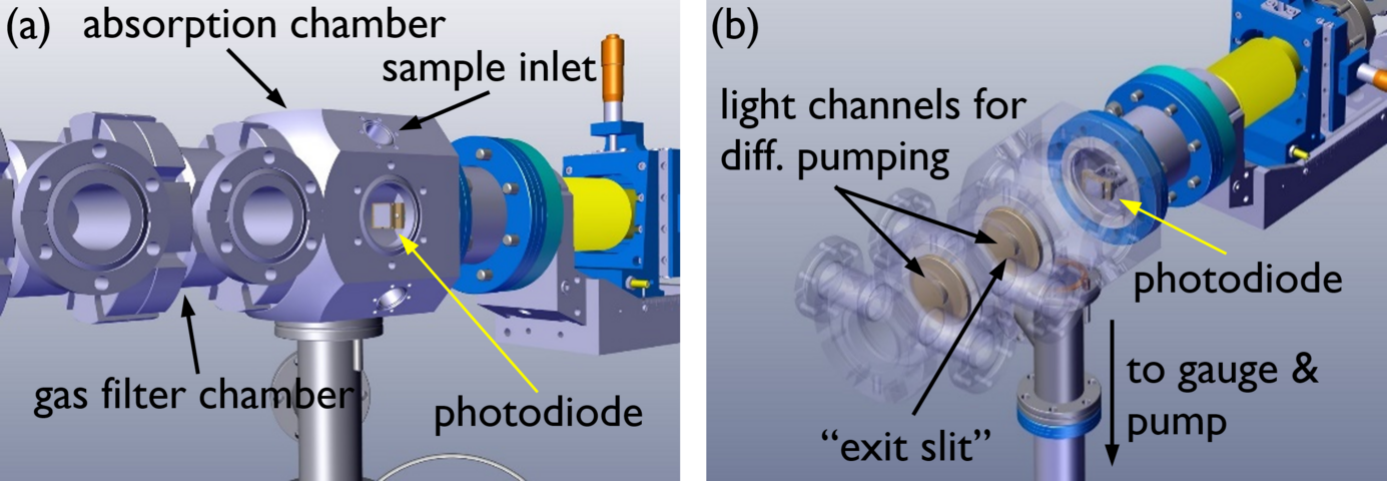
The adapted differently pumped rare-gas filter shown with the new absorption chamber. The gas filter chamber is filled with 10 mbar of rare gas to absorb the higher-harmonic radiation of the grating. The photodiode detector is mounted on an *xyz*-manipulator. The copper plates with the differential pumping apertures are seen in the semi-transparent representation in (*b*).

The bending magnet synchrotron radiation of the VUV beamline at the SLS was collimated, dispersed using a 150 lines mm^−1^ laminar grating, and focused ∼22 m from the source point of the bending magnet (Bodi *et al.*, 2012 ▸). Normally, the focus is at the variable exit slit in a cell downstream from the gas inlet chamber in the rare-gas filter, after which the gas filter is interfaced to a photoelectron photoion coincidence endstation by two further differential pumping stages (Sztáray *et al.*, 2017 ▸; Johnson *et al.*, 2009 ▸). In the VUV absorption experiments, we removed the last three differentially pumped chambers of the rare-gas filter towards the endstation port to have optical access and flexibility in terms of the absorbing medium. The chamber with the focus, originally housing the exit slit, has been replaced by the absorption chamber as seen in Fig. 1 ▸. Thus, with respect to the original setup, the last remaining gas filter chamber is the rare-gas inlet, which was filled with an Ne–Ar–Kr mixture or pure Ne, depending on the photon energy range of interest, to provide high-harmonic-free radiation in the absorption chamber. The absorption chamber is connected by a 1 mm channel through a precision-made copper plate, as seen in Fig. 1 ▸(*b*), with the rare-gas inlet chamber. This channel also acted as the exit slit. Setting up the beamline for absorption experiments requires ∼3 h of time. We used the Ar absorption edge in the first and second order as well as the Kr absorption edge in the first order to calibrate the photon energy scale and determine the photon energy resolution. The latter was found to be 10 meV at 7 eV and 20–30 meV in the 14–16 eV range, based on the full width at half-maximum of the derivative of the Ar and Kr absorption edges. The resolving power could be further increased about fivefold at the cost of photon flux by installing a 200 µm exit slit in the center of the absorption chamber at the focus of the beamline.

Equilibrium vacuum conditions are quickly reached when the pressure is highest in the rare-gas chamber. The gas filter can work at filter gas pressures up to 30 mbar, which means the maximum pressure in the absorption cell can be 25 mbar without risking sample diffusion into the filter cell. In the current measurements, the filter gas pressure was measured at 9.9 mbar using a capacitance gauge, so that there is filter gas flow towards the absorption chamber. The absorption chamber was pumped using an ACP 120G Roots pump through a 40 mm VAT diaphragm valve to reduce pumping speed. The pressure in the absorption chamber was set using a needle valve for the sample inlet and the diaphragm valve to control pumping. The background pressure with the gas filter on was measured by a Pirani gauge connected close to the chamber at the pumping line to be ∼0.2 mbar, whereas sample absorption measurements were taken at pressure readings of 0.6–1.1 mbar for H_2_O and 0.36–0.42 mbar for N_2_O. Because of the limited accuracy of the Pirani pressure readings as well as the difficult-to-characterize contributions of the rare-gas diffusion into the absorption chamber, the column density of the sample is unknown, and overlapping scans and low-energy cross sections reported in the literature were used to anchor the measured extinctions to absolute absorption cross sections.

The light was detected using an SXUV100 photodiode as seen in Fig. 1 ▸(*b*), and the resulting photocurrent was measured using a Keithley 6485 picoammeter. In each scan, the photocurrent was averaged for 1–2 s at each photon energy, which was scanned in a 5–50 meV step size. The beamline transmission was measured with a running gas filter but without sample in between scans using comparable settings. The flux curve of the beamline was found to drift only slightly during the measurement campaign. Reference scans before and after absorption scans were used to obtain the extinction. The sample pressure was set so that the extinction did not exceed 90% and averaged around 50% across the spectral range, to ensure the linearity of the photocurrent with respect to the photon flux.

## 3. Results and discussion

**Figure 2:**
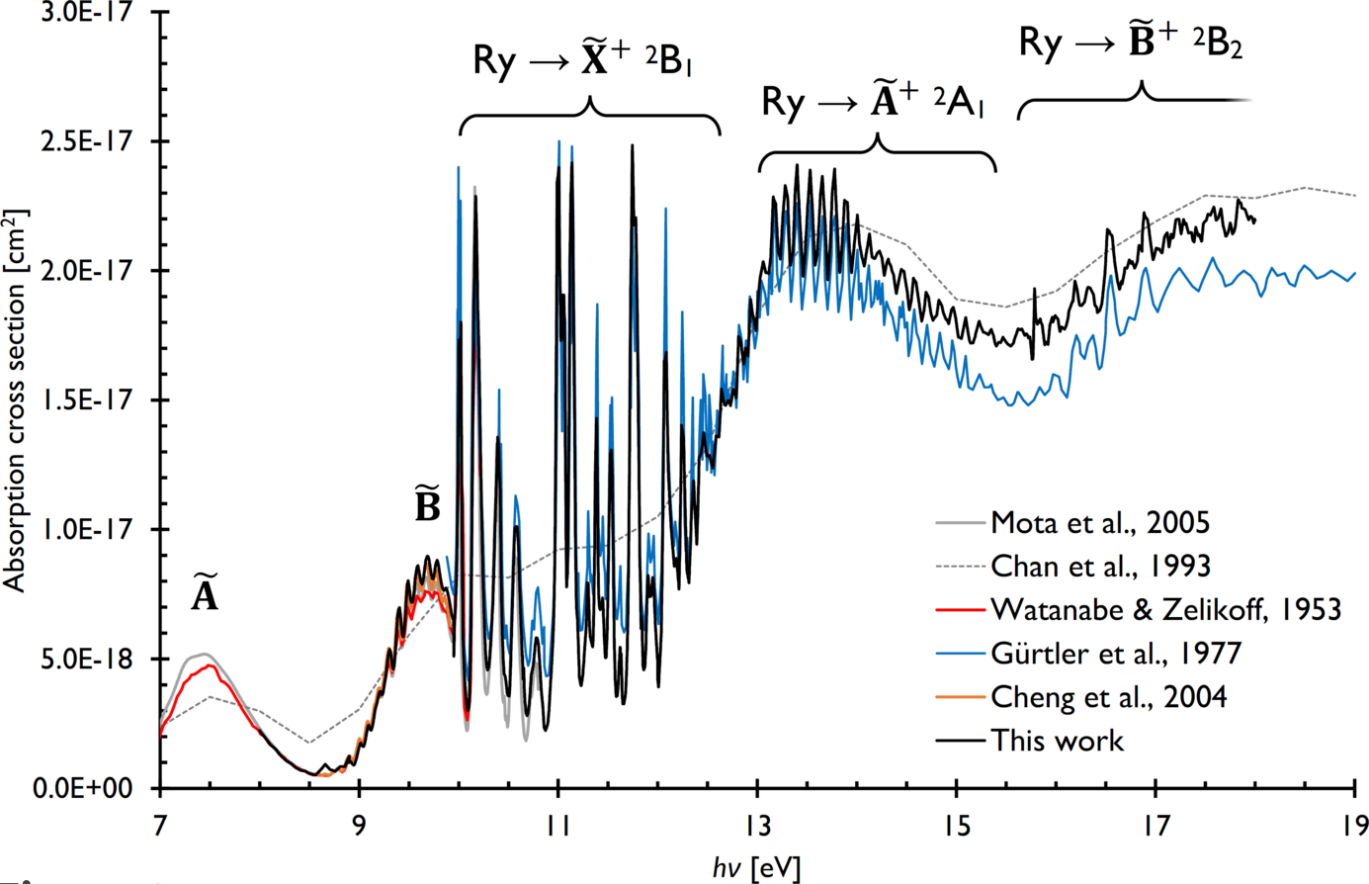
The newly recorded VUV absorption spectrum of water in the 8–18 eV energy range shown together with previous results and the low-resolution, broad-range (e,e) spectrum by Chan *et al.* (1993 ▸).

Absorption spectra were recorded in the 8–18 eV and 7.5–19 eV range for water and nitrous oxide and are shown in Figs. 2 ▸ and 3 ▸, respectively, together with previous measurements as compiled in the MPI-Mainz UV/VIS Spectral Atlas, with the cross sections occasionally converted from the originally published absorption coefficients by the Atlas (Keller-Rudek *et al.*, 2013 ▸). The newly recorded absorption spectra are also available in the supporting information.

The water absorption spectrum has been studied quite extensively. As determined by the working range of the differentially pumped gas filter, our energy range starts at ∼7 eV and reaches up to 20 eV, where characteristic cross sections are of the order of 10^−17^ cm^2^, *i.e.* 10 Mb, as shown in Fig. 2 ▸ together with previous absorption measurements and the relevant part of the (e,e) spectrum of water, recorded between 6 and 200 eV by Chan *et al.* (1993 ▸). The ∼1 eV resolution of the water (e,e) spectrum only depicts changes in the average absorption cross section and does not reveal the rich structure of the spectrum.

This is best seen in the synchrotron-based measurement of Gürtler *et al.* (1977 ▸), who recorded cross sections in the 10–60 eV range. The structure seen between 10 and 12.6 eV can be assigned to 3–5*p* and 3–6*d* Rydberg states as well as their vibrational fine structure, converging to the ionization threshold to the ground 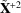
^2^B_1_ cation state at 12.617 eV (Ruscic, 2023 ▸). The onset of the first excited 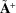
^2^A_1_ cation state was reported at 13.748 eV by Truong *et al.* (2009 ▸), but the corresponding vertical ionization energy is 14.8 eV. This is also where the *ns* and *np* Rydberg progressions, giving rise to the vibrational fine structure in the 13–16 eV energy range in the absorption spectrum, were assumed to converge to by Gürtler *et al.* The next, 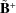
^2^B_2_ band in the photoelectron spectrum appears at 17.203 eV (Truong *et al.*, 2009 ▸), exhibits a vertical ionization energy of ∼18.5 eV, and contains the dissociative ionization onset at 18.118 eV (Bodi *et al.*, 2014 ▸) with a corresponding ∼fourfold jump in the threshold ionization cross section at unchanging absorption cross sections, *i.e.* corresponding to a jump in the threshold ionization quantum efficiency at this energy (Truong *et al.*, 2009 ▸; Bodi *et al.*, 2014 ▸). The vibrational fine structure in the absorption spectrum above 16 eV is due to the Rydberg progression towards the 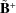 state.

Turning attention towards lower energies, we highlight three prior results. Watanabe & Zelikoff used a hydrogen discharge tube and reported the absorption of water vapor in the 6.7–10.2 eV photon energy range in their seminal paper (Watanabe & Zelikoff, 1953 ▸). More recently, Mota *et al.* (2005 ▸) measured light water, whereas Cheng *et al.* (2004 ▸) measured light and heavy water absorption using synchrotron radiation in the 6.4–10.8 eV and 8.55–9.92 eV energy ranges, respectively. Mota *et al.*’s spectrum shows significant overlap with that of Gürtler *et al.*, and all three lower-energy scans can be used to anchor our spectrum to obtain absolute absorption cross sections across the complete energy range studied herein. The band centered around 7.5 eV is the 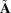 band of the water spectrum and corresponds to a 3*s* ← *b*_1_ excitation belonging to the Rydberg states converging to the cation ground state, whereas excitation of an *a*_1_ electron to the same 3*s* orbital results in the second 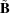 band of the water VUV spectrum, centered at 9.7 eV and belonging to the progression converging to the excited 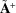
^2^A_1_ cation state.

The evident fine structure of the 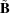 band sitting on top of an intense and broad baseline has fascinated researchers for a long time (van Harrevelt *et al.*, 2001 ▸). While the assignment of the normal modes giving rise to this structure is straightforward (Mota *et al.*, 2005 ▸), it falls short of capturing the double nature of this band as well as the subtle effects of the different potential energy wells as the energy rises above the HOH ↔ HHO barrier (Cheng *et al.*, 2004 ▸). Isotopologue measurements and Monte Carlo quantum chemical calculations have conclusively established the role of resonances responsible for the fine structure as being due to the HOH well and are well described by bending and stretching vibrations below 9 eV, and to the HOH well coupled by quantum tunneling to the HHO well slightly above 9 eV, and again simply described well above 9 eV. Furthermore, in contrast to the resonances, the broad background is due to fast coupling to and dissociation on the ground state, as also illustrated by the different electronic states of the OH radicals produced (Zhou *et al.*, 2015 ▸).

The newly recorded absorption spectrum, fitted to the literature values around 9 eV to obtain absolute cross sections, generally agrees with the previously measured ones across the studied energy range in terms of cross sections and resonance energies. The resonances reported by Gürtler *et al.* are sharper, thanks to the higher energy resolution of their light source, but this could be reproduced in our experiment, as well, with the help of a well positioned exit slit. The only notable but minor difference is seen above 14 eV, where our cross sections are, on average, ∼13% larger than those reported by Gürtler *et al.* (1977 ▸) and agree slightly better with the (e,e) data of Chan *et al.* (1993 ▸).

**Figure 3:**
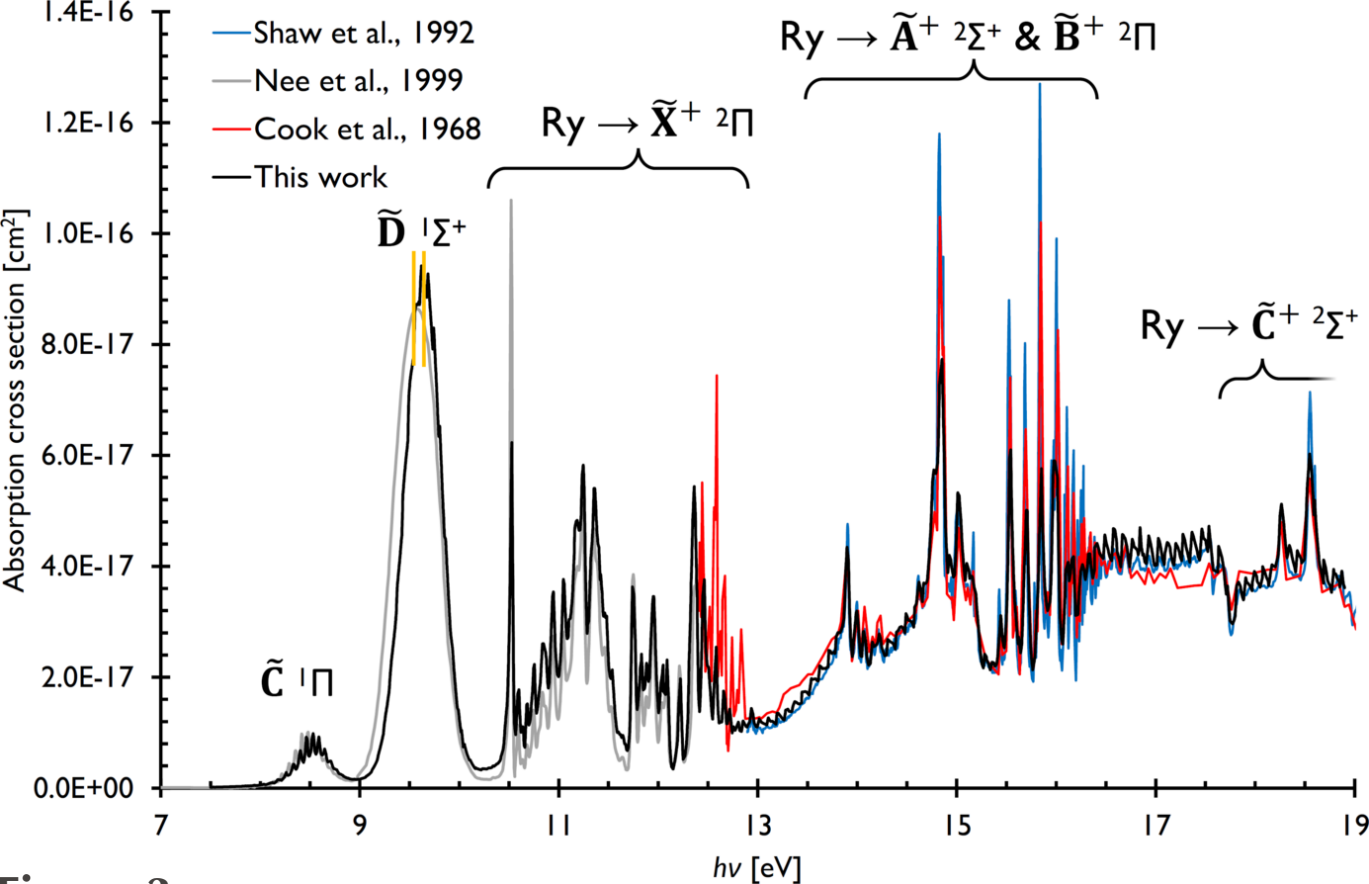
The newly recorded VUV absorption spectrum of nitrous oxide in the 7.5–19.0 eV photon energy range shown together with previous results. The orange lines indicate the small shift in the 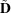 band with respect to the spectrum of Nee *et al.* (1999 ▸).

The valence cation states of nitrous oxide have been studied using He I photoelectron spectroscopy (Brundle & Turner, 1969 ▸) and photoionization mass spectrometry (Berkowitz & Eland, 1977 ▸). Employing ion internal energy selection, Guyon *et al.* studied interactions between neutral dissociation and ionization continua after valence photoionization (Guyon *et al.*, 1983 ▸). They also estimated the photoionization quantum efficiency at 15.5 eV to be constant at ∼0.5, which was later shown to oscillate quite strongly around 0.7 in this energy range (Shaw *et al.*, 1992 ▸). Ionization from the 2π, 7σ and 1π HOMO, HOMO-1 and HOMO-2 orbitals of N_2_O is known to result in the first three 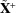
^2^Π, 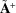
^2^Σ^+^ and 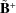
^2^Π cation electronic states at ionization energies of 12.886, 16.388 and 17.65 eV, respectively. These ionization limits must be considered when interpreting the nitrous oxide absorption spectrum, shown together with literature results in Fig. 3 ▸.

Cook *et al.* used a Hopfield-continuum light source and recorded the spectrum in the 12.4–20.7 eV energy range (Cook *et al.*, 1968 ▸), to be complemented by Nee *et al.* between 5.6 and 12.4 eV (Nee *et al.*, 1999 ▸) using a synchrotron light source and a LiF windowed cell to obtain well defined column densities and absolute cross sections in the lower energy range. We relied on these to normalize our absorption spectrum to obtain absolute cross sections, as well. As previously mentioned, Shaw *et al.*’s ultimate goal was to report photoionization efficiencies, for which they recorded absolute absorption cross sections upwards from the ionization onset in the 12.9–25.6 eV energy range at the SRS in Daresbury (Shaw *et al.*, 1992 ▸). Furthermore, high-resolution absorption spectra were reported by Cossart-Magos *et al.* in the 9.3–12.9 eV energy range (Cossart-Magos *et al.*, 2001 ▸), while Duflot *et al.* and Jones reported and compared a high-resolution N_2_O (e,e) spectrum and an absorption spectrum (Duflot *et al.*, 2019 ▸; Jones, 2000 ▸).

The dipole forbidden 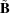
^1^Δ band of N_2_O is broad, weak with a maximum cross section of 1.4 × 10^−19^ cm^2^, centered at 6.85 eV, below our region of interest. It is followed by the dissociative 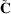
^1^Π band (Yuan *et al.*, 2018 ▸), exhibiting vibrational fine structure of ∼1000 cm^−1^ around 8.5 eV. The 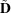
^1^Σ^+^ band, centered at 9.5 eV or slightly higher, was previously described as featureless (Nee *et al.*, 1999 ▸), although some fine structure is evident in Fig. 3 ▸. Zelikoff *et al.* reported a number of diffuse bands in the 9.3–9.6 eV energy range, which are tentatively visible in our data, as well (Zelikoff *et al.*, 1953 ▸). However, they have not observed fine structure on the blue side of the band, where it appears to be more apparent in our spectrum. The 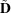
^1^Σ^+^ band is also dissociative, the mechanism of which, including the participation of the nearby ^3^Π_v_ state, was discussed by Lambert *et al.* (2005 ▸). The next sharp and strong feature in the spectrum, observed at 10.521 eV, was previously assigned to the 2π^3^5π ^1^Σ^+^ state (Hopper, 1984 ▸), later revised as a vibrationally excited 3*p*σ ^1^Π Rydberg state (Szarka & Wallace, 1991 ▸), then as a vibrationally ground state of the same configuration (Cossart-Magos *et al.*, 2001 ▸). Although they have not reported absorption cross sections, Cossart-Magos *et al.* have thoroughly assigned the peaks below 12.9 eV to various Rydberg progressions converging to the 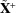, 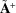 and 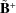 cation states (Cossart-Magos *et al.*, 2001 ▸). It is also in this energy range, between 12.5 and 13.0 eV, that the absorption cross sections of Cook *et al.* deviate the most from our results (Cook *et al.*, 1968 ▸). The results of Cossart-Magos *et al.* and Jones agree with our band profile in this energy region, and, based on the discussion of the former, it is possible that a nitro­gen impurity led to the skewed band profile in the spectrum of Cook *et al.* (1968 ▸) just below 13 eV (Duflot *et al.*, 2019 ▸; Jones, 2000 ▸; Cossart-Magos *et al.*, 2001 ▸).

At 12.886 eV, we reach the ionization energy to the 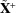
^2^Π state, which coincides with the onset of a continuum-like absorption region with monotonously rising cross sections up to 13.9 eV. While neither Shaw *et al.* nor Cook *et al.* reported a fine structure in this energy region, the photoabsorption data shown by Duflot *et al.* exhibit some peaks. Our data, confirmed by the raw photocurrent profile over three scans in this energy range, is suggestive of a stepwise increase of the cross sections, indicative of a vibrational structure and a coupled continuum state responsible for the rising baseline. There are no cation final states in this energy region, and the photoionization quantum efficiency is monotonously dropping (Shaw *et al.*, 1992 ▸). Thus, it is likely that neutral final states are responsible for the rise in the photoabsorption cross section. This band comes to an end with four to five peaks in the 13.89–14.22 eV range, assigned to vibrationally ground and excited 3*p*σ and 3*p*π Rydberg states converging to the 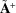
^2^Σ^+^ and 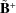
^2^Π cation states, respectively. Thereafter, the spectrum is dominated by excitation into *np*σ, *ns*σ, *nd*π and *nd*σ states up to the 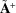
^2^Σ^+^ ionization energy at 16.39 eV. According to Shaw *et al.* (1992 ▸), the photoionization quantum efficiency anticorrelates with the photoabsorption cross section in this energy range, indicative of neutral decay channels. The fine structure at 16.5–17.8 eV, in part overlapping with the 17.65 eV onset of the broad and resolved 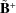
^2^Π cation state band in the photoelectron spectrum (Brundle & Turner, 1969 ▸), has also been observed by a more recent measurement on N_2_O photodissociation by Shaw & Holland (2008 ▸) and is somewhat better resolved by the current measurement. It is likely due to Rydberg progressions to this cation state. The sharp peaks at 18.23 and 18.56 eV, also reported by Shaw *et al.* (1992 ▸), must be due to Rydberg states belonging to the manifold converging to the 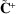
^2^Σ^+^ cation state at 20.11 eV.

Overall, the newly recorded spectrum agrees well with the literature data, although we report the maximum of the 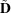 band at a slightly higher energy with a hint of a superimposed fine structure beyond the literature results. Based also on more recent measurements, we also propose that the band shape below 13 eV, as recorded by Cook *et al.*, was likely affected by impurities. Furthermore, oscillations in the spectrum slightly above 13 eV are newly and around 17 eV somewhat more clearly resolved by the current measurements compared with the literature.

## 4. Conclusions

Based on a differentially pumped rare-gas filter, we constructed an absorption cell to be used in the 7–21 eV energy range to measure the absorption cross sections of gaseous samples. The absorption spectrum of water and nitrous oxide were recorded in commissioning experiments in less than 12 h. Both spectra span most of the VUV valence excitation and ionization region of the samples and represent a novel avenue to cover such a broad energy range (7–21 eV, 170–59 nm or 56000–170000 cm^−1^) in one go. The spectra were calibrated using literature measurements with known column densities to obtain absolute photoabsorption cross sections, which generally agreed with literature results very well, while also revealing new details in the absorption spectrum of N_2_O.

These commissioning experiments have also highlighted four avenues, along which the experiment could be developed further. First, more than half of the measurement time was spent waiting for the monochromator to change energy and come to a stop. In light of the short photocurrent averaging times, data acquisition could therefore be speeded up considerably by implementing continuous, on-the-fly scanning instead of the point-to-point stop-and-go approach employed currently at the beamline (Lin *et al.*, 2013 ▸). Second, by building an MgF_2_- or LiF-windowed gas cell, we could carry out the calibration experiments to determine absolute cross sections to which the differentially pumped setup can be calibrated to. Third, by installing an exit slit or exit skimmer at the focus of the chamber, we could reach the nominal resolution of the beamline, *i.e.E*/Δ*E* of up to 1000–10000, depending on the grating and the photon energy. Fourth, some of the newly, tentatively observed structure in the N_2_O spectrum could likely be made more evident by cooling down the sample, thereby sharpening the rotational envelope.

In summary, this setup will allow for VUV absorption measurements of gaseous samples above the absorption edge of MgF_2_ and LiF. Our absorption experiment will fill a gap in this regard, as most light sources and experiments capable of such measurements have been decommissioned in recent years. The chamber is also designed to allow for condensed phase absorption measurements in this energy range, such as liquid or solid thin films. This also opens up the possibility to determine the absorbance of condensed-phase samples in the VUV.

## Supplementary Material

Absorption spectra in Excel format. DOI: 10.1107/S1600577524005423/rv5179sup1.xlsx
